# 4-Amino-3-(*o*-tolyl­oxymeth­yl)-1*H*-1,2,4-triazole-5(4*H*)-thione

**DOI:** 10.1107/S1600536809027275

**Published:** 2009-07-18

**Authors:** Hoong-Kun Fun, Wei-Ching Liew, A. M. Vijesh, Mahesh Padaki, Arun M. Isloor

**Affiliations:** a X-ray Crystallography Unit, School of Physics, Universiti Sains Malaysia, 11800 USM, Penang, Malaysia; b SeQuent Scientific Limited, No. 120 A&B, Industrial Area, Baikampady, New Mangalore, Karnataka 575 011, India; c Department of Chemistry, National Institute of Technology-Karnataka, Surathkal, Mangalore 575 025, India

## Abstract

The asymmetric unit of the title compound, C_10_H_12_N_4_OS, contains two independent mol­ecules, *A* and *B*, which differ significantly in the relative orientations of the benzene and triazole rings. The dihedral angle between the above two rings is 6.94 (5)° in mol­ecule *A* and 77.60 (5)° in mol­ecule *B*. In the crystal, mol­ecules are linked into a three-dimensional network by N—H⋯S, N—H⋯O, N—H⋯N and C—H⋯S hydrogen bonds and π–π inter­actions between the benzene and triazole rings [centroid–centroid distance = 3.5311 (6) Å] are also present.

## Related literature

For the pharmaceutical activity of triazole derivatives, see: Amir *et al.* (2008 ▶); Kuş *et al.* (2008 ▶); Padmavathi *et al.* (2008 ▶); Sztanke *et al.* (2008 ▶). For the preparation, see: Eweiss *et al.* (1986 ▶). For bond-length data, see: Allen *et al.* (1987 ▶). For related structures, see: Fun *et al.* (2008**a* ▶,b* ▶, 2009 ▶). For the stability of the temperature controller used in the data collection, see: Cosier & Glazer (1986 ▶).
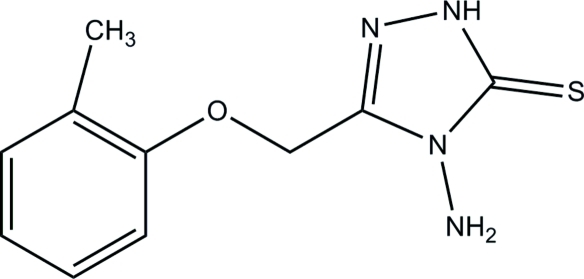


## Experimental

### 

#### Crystal data

C_10_H_12_N_4_OS
*M* *_r_* = 236.30
Orthorhombic, 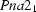

*a* = 8.6908 (1) Å
*b* = 22.2551 (3) Å
*c* = 11.3771 (2) Å
*V* = 2200.50 (5) Å^3^
*Z* = 8
Mo *K*α radiation
μ = 0.28 mm^−1^
*T* = 100 K
0.58 × 0.29 × 0.27 mm

#### Data collection

Bruker SMART APEXII CCD area-detector diffractometer
Absorption correction: multi-scan (**SADABS**; Bruker, 2005 ▶) *T*_min_ = 0.855, *T*_max_ = 0.929
41442 measured reflections
9726 independent reflections
9145 reflections with *I* > 2σ(*I*)
*R*_int_ = 0.029

#### Refinement

*R*[*F* ^2^ > 2σ(*F* ^2^)] = 0.028
*wR*(*F* ^2^) = 0.075
*S* = 1.01
9726 reflections
315 parameters
1 restraint
H atoms treated by a mixture of independent and constrained refinement
Δρ_max_ = 0.33 e Å^−3^
Δρ_min_ = −0.19 e Å^−3^
Absolute structure: Flack (1983 ▶), 4628 Friedel pairs
Flack parameter: −0.02 (3)

### 

Data collection: *APEX2* (Bruker, 2005 ▶); cell refinement: *SAINT* (Bruker, 2005 ▶); data reduction: *SAINT*; program(s) used to solve structure: *SHELXTL* (Sheldrick, 2008 ▶); program(s) used to refine structure: *SHELXTL*; molecular graphics: *SHELXTL*; software used to prepare material for publication: *SHELXTL* and *PLATON* (Spek, 2009 ▶).

## Supplementary Material

Crystal structure: contains datablocks global, I. DOI: 10.1107/S1600536809027275/ci2852sup1.cif

Structure factors: contains datablocks I. DOI: 10.1107/S1600536809027275/ci2852Isup2.hkl

Additional supplementary materials:  crystallographic information; 3D view; checkCIF report

## Figures and Tables

**Table 1:** Hydrogen-bond geometry (Å, °)

*D*—H⋯*A*	*D*—H	H⋯*A*	*D*⋯*A*	*D*—H⋯*A*
N4*A*—H1*N*4⋯N4*B*^i^	0.88 (2)	2.46 (2)	3.2651 (12)	152 (2)
N4*A*—H2*N*4⋯O1*B*	0.83 (2)	2.53 (2)	3.3560 (11)	171 (2)
N4*B*—H4*N*4⋯S1*A*^ii^	0.95 (2)	2.72 (2)	3.6167 (10)	157 (2)
N2*A*—H2*N*1⋯S1*B*^iii^	0.87 (2)	2.30 (2)	3.1665 (9)	174 (2)
N2*B*—H2*N*2⋯N1*A*^iv^	0.89 (2)	2.18 (2)	3.0589 (11)	166 (2)
C8*A*—H8*AA*⋯S1*A*^v^	0.93	2.86	3.4537 (10)	123
C3*B*—H3*BB*⋯S1*A*	0.97	2.86	3.8203 (10)	170

Symmetry codes: (i) 
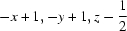
; (ii) 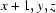; (iii) 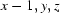; (iv) 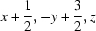; (v) 
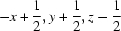
.

**Fig. 1.**
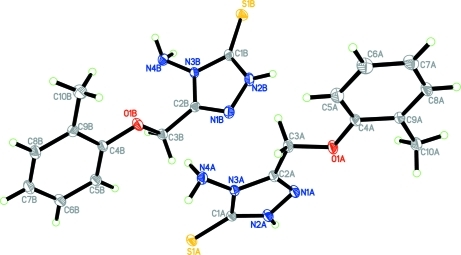
The asymmetric unit of the title compound, showing 50% probability displacement ellipsoids and the atom-numbering scheme.

**Fig. 2.**
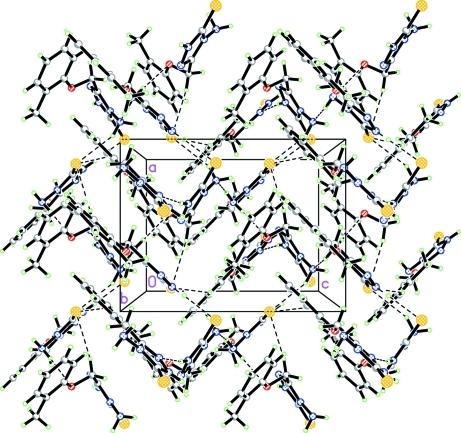
The three-dimensional network of the title compound, viewed along the b axis. Hydrogen bonds are shown as dashed lines.

## supplementary crystallographic information

### Comment

1,2,4-Triazole and its derivatives were reported to exhibit various pharmacological activities such as antimicrobial, analgesic, anticancer, anti-inflammatory and antioxidant properties (Amir *et al.*, 2008; Kuş *et al.*, 2008; Padmavathi *et al.*, 2008; Sztanke *et al.*, 2008). Some of the present day drugs such as ribavirin (antiviral agent), rizatriptan (antimigraine agent), alprazolam (anxiolytic agent), fluconazole and itraconazole (antifungal agents) are the best examples for potent molecules possessing triazole nucleus. The amino and mercapto groups of 1,2,4-triazoles serve as readily accessible nucleophilic centers for the preparation of N-bridged heterocycles. Keeping in view of this biological importance, the title compound was synthesized and its crystal structure is reported here.

In the title compound (Fig. 1), the bond lengths (Allen *et al.*, 1987) and angles are found to have normal values and are comparable to closely related structures (Fun *et al.*, 2008a,b,2009). The dihedral angle between the triazole ring (N1A-N3A/C1A/C2A) and the benzene ring (C4A-C9A) of molecule *A* is 6.94 (5)°, whereas the dihedral angle between the triazole ring (N1B—N3B/C1B/C2B) and the benzene ring (C4B—C9B) of molecule *B* is 77.60 (5)° indicating that for molecule *B*, these rings are significantly twisted from each other.

The crystal packing (Fig. 2) is consolidated by N—H···S, N—H···O, N—H···N and C—H···S hydrogen bonds, linking the molecules into a three-dimensional network (Table 1). The crystal packing is further strengthened by π-π interactions between the N1A-N3A/C1A/C2A (centroid *Cg*1) ring of molecule *A* at (x, y, z) and C4A-C9A (centroid *Cg*2) ring of molecule *A* at (x-1/2, 3/2-y, z), with a centroid-to-centroid distance of 3.5311 (6) Å.

### Experimental

*O*-Cressoyloxyacetyl hydrazine (18.0 g, 0.1 mol) was added slowly to a solution of potassium hydroxide (8.4 g, 0.15 mol) in ethanol (150 ml). The resulting mixture was stirred well till a clear solution was obtained. Carbon disulfide (11.4 g, 0.15 mol) was added drop-wise and the contents were stirred vigorously. Further stirring was continued for 24 h. The resulting mixture was diluted with 100 ml of ether and the precipitate formed was collected by filtration, washed with dry ether and dried at 65 °C under vacuum. It was used for the next step without any purification.

A mixture of potassium dithiocarbazinate (29.4 g, 0.1 mol), hydrazine hydrate (99%, 0.2 mol) and water (2 ml) was gently heated to boil for 30 minutes. Heating was continued until the evacuation of hydrogen sulfide ceases. The reaction mixture was cooled to room temperature, diluted with water (100 ml) and acidified with HCl. The solid mass that separated was collected by filtration, washed with water and dried. Recrystallization was done from ethanol. Yield: 13.7 g, 58.0%, *m.p.* 400–402 K (Eweiss *et al.*, 1986).

### Refinement

N-bound H atoms were located in a difference Fourier map and refined freely. C-bound H atoms were positioned geometrically [C-H = 0.93–0.97 Å] and refined using a riding model, with *U*_iso_(H) = 1.2*U*_eq_(C) and 1.5*U*_eq_(methyl C). A rotating group model was used for the methyl groups.

### Crystal data

**Table:**

C_10_H_12_N_4_OS	*F*(000) = 992
*M**_r_* = 236.30	*D*_x_ = 1.427 Mg m^−^^3^
Orthorhombic, *P**n**a*2_1_	Mo *K*α radiation, λ = 0.71073 Å
Hall symbol: P 2c -2n	Cell parameters from 9872 reflections
*a* = 8.6908 (1) Å	θ = 2.5–35.1°
*b* = 22.2551 (3) Å	µ = 0.28 mm^−^^1^
*c* = 11.3771 (2) Å	*T* = 100 K
*V* = 2200.50 (5) Å^3^	Block, colourless
*Z* = 8	0.58 × 0.29 × 0.27 mm

### Data collection

**Table:**

Bruker SMART APEXII CCD area-detector diffractometer	9726 independent reflections
Radiation source: fine-focus sealed tube	9145 reflections with *I* > 2σ(*I*)
graphite	*R*_int_ = 0.029
φ and ω scans	θ_max_ = 35.2°, θ_min_ = 1.8°
Absorption correction: multi-scan (*SADABS*; Bruker, 2005)	*h* = −14→14
*T*_min_ = 0.855, *T*_max_ = 0.929	*k* = −35→35
41442 measured reflections	*l* = −18→18

### Refinement

**Table:**

Refinement on *F*^2^	Secondary atom site location: difference Fourier map
Least-squares matrix: full	Hydrogen site location: inferred from neighbouring sites
*R*[*F*^2^ > 2σ(*F*^2^)] = 0.028	H atoms treated by a mixture of independent and constrained refinement
*wR*(*F*^2^) = 0.075	*w* = 1/[σ^2^(*F*_o_^2^) + (0.0433*P*)^2^ + 0.2689*P*] where *P* = (*F*_o_^2^ + 2*F*_c_^2^)/3
*S* = 1.01	(Δ/σ)_max_ = 0.002
9726 reflections	Δρ_max_ = 0.33 e Å^−^^3^
315 parameters	Δρ_min_ = −0.19 e Å^−^^3^
1 restraint	Absolute structure: Flack (1983), 4628 Friedel pairs
Primary atom site location: structure-invariant direct methods	Flack parameter: −0.02 (3)

### Special details

**Table:**

Experimental. The crystal was placed in the cold stream of an Oxford Cyrosystems Cobra open-flow nitrogen cryostat (Cosier & Glazer, 1986) operating at 100.0 (1) K.
Geometry. All e.s.d.'s (except the e.s.d. in the dihedral angle between two l.s. planes) are estimated using the full covariance matrix. The cell e.s.d.'s are taken into account individually in the estimation of e.s.d.'s in distances, angles and torsion angles; correlations between e.s.d.'s in cell parameters are only used when they are defined by crystal symmetry. An approximate (isotropic) treatment of cell e.s.d.'s is used for estimating e.s.d.'s involving l.s. planes.
Refinement. Refinement of *F*^2^ against ALL reflections. The weighted *R*-factor *wR* and goodness of fit *S* are based on *F*^2^, conventional *R*-factors *R* are based on *F*, with *F* set to zero for negative *F*^2^. The threshold expression of *F*^2^ > σ(*F*^2^) is used only for calculating *R*-factors(gt) *etc*. and is not relevant to the choice of reflections for refinement. *R*-factors based on *F*^2^ are statistically about twice as large as those based on *F*, and *R*- factors based on ALL data will be even larger.

### Fractional atomic coordinates and isotropic or equivalent isotropic displacement parameters (Å^2^)

**Table:**

	*x*	*y*	*z*	*U*_iso_*/*U*_eq_	
S1A	0.08512 (3)	0.532701 (9)	0.19019 (2)	0.01524 (4)	
O1A	0.33838 (9)	0.77054 (3)	−0.00794 (7)	0.01789 (13)	
N1A	0.18273 (11)	0.70334 (3)	0.15180 (7)	0.01539 (14)	
N2A	0.10587 (10)	0.65525 (3)	0.20140 (8)	0.01580 (14)	
N3A	0.25603 (10)	0.61737 (3)	0.07393 (7)	0.01237 (13)	
N4A	0.34200 (11)	0.57827 (3)	0.00333 (7)	0.01508 (14)	
C1A	0.14799 (11)	0.60200 (4)	0.15681 (8)	0.01298 (14)	
C2A	0.27309 (11)	0.67851 (4)	0.07402 (8)	0.01336 (15)	
C3A	0.38198 (12)	0.70933 (4)	−0.00655 (9)	0.01540 (15)	
H3AA	0.4868	0.7051	0.0215	0.018*	
H3AB	0.3754	0.6923	−0.0849	0.018*	
C4A	0.42434 (11)	0.80824 (4)	−0.07765 (9)	0.01387 (14)	
C5A	0.53343 (13)	0.78880 (4)	−0.15850 (9)	0.01853 (17)	
H5AA	0.5524	0.7480	−0.1685	0.022*	
C6A	0.61434 (14)	0.83133 (5)	−0.22470 (10)	0.02183 (19)	
H6AA	0.6870	0.8188	−0.2795	0.026*	
C7A	0.58630 (13)	0.89233 (5)	−0.20862 (9)	0.02055 (19)	
H7AA	0.6413	0.9207	−0.2516	0.025*	
C8A	0.47564 (13)	0.91072 (4)	−0.12798 (9)	0.01740 (17)	
H8AA	0.4571	0.9516	−0.1180	0.021*	
C9A	0.39210 (11)	0.86951 (4)	−0.06189 (8)	0.01362 (15)	
C10A	0.27083 (13)	0.88901 (4)	0.02363 (10)	0.01894 (18)	
H10A	0.2761	0.9318	0.0340	0.028*	
H10B	0.2875	0.8695	0.0978	0.028*	
H10C	0.1712	0.8783	−0.0061	0.028*	
S1B	0.89572 (3)	0.691199 (11)	0.41761 (2)	0.01782 (5)	
O1B	0.57901 (9)	0.49790 (3)	0.17514 (7)	0.01652 (13)	
N1B	0.53427 (11)	0.63533 (4)	0.24499 (8)	0.01717 (15)	
N2B	0.63760 (11)	0.67783 (4)	0.28244 (8)	0.01748 (15)	
N3B	0.71458 (10)	0.59546 (3)	0.35636 (7)	0.01283 (13)	
N4B	0.79643 (11)	0.55068 (4)	0.41643 (9)	0.01770 (14)	
C1B	0.74960 (12)	0.65567 (4)	0.35097 (8)	0.01391 (15)	
C2B	0.58365 (11)	0.58530 (4)	0.29216 (8)	0.01315 (15)	
C3B	0.50974 (11)	0.52552 (4)	0.27590 (8)	0.01388 (15)	
H3BA	0.5256	0.5008	0.3450	0.017*	
H3BB	0.3999	0.5302	0.2637	0.017*	
C4B	0.53275 (11)	0.43946 (4)	0.15063 (8)	0.01406 (15)	
C5B	0.40519 (12)	0.41259 (4)	0.20300 (9)	0.01678 (16)	
H5BA	0.3470	0.4336	0.2579	0.020*	
C6B	0.36530 (13)	0.35369 (4)	0.17238 (9)	0.01886 (18)	
H6BA	0.2794	0.3357	0.2060	0.023*	
C7B	0.45408 (14)	0.32217 (4)	0.09177 (9)	0.01915 (18)	
H7BA	0.4287	0.2828	0.0723	0.023*	
C8B	0.58097 (13)	0.34962 (4)	0.04025 (9)	0.01721 (17)	
H8BA	0.6396	0.3281	−0.0137	0.021*	
C9B	0.62274 (12)	0.40874 (4)	0.06744 (8)	0.01428 (15)	
C10B	0.75914 (14)	0.43787 (5)	0.00943 (9)	0.02039 (18)	
H10D	0.8209	0.4077	−0.0280	0.031*	
H10E	0.8195	0.4584	0.0676	0.031*	
H10F	0.7241	0.4662	−0.0483	0.031*	
H1N4	0.281 (2)	0.5525 (8)	−0.0322 (16)	0.028 (4)*	
H2N4	0.403 (2)	0.5622 (7)	0.0500 (16)	0.024 (4)*	
H3N4	0.811 (2)	0.5664 (7)	0.4897 (18)	0.034 (4)*	
H4N4	0.892 (2)	0.5482 (9)	0.3764 (18)	0.038 (5)*	
H2N1	0.050 (2)	0.6623 (8)	0.2633 (17)	0.029 (4)*	
H2N2	0.638 (2)	0.7148 (8)	0.2518 (16)	0.028 (4)*	

### Atomic displacement parameters (Å^2^)

**Table:**

	*U*^11^	*U*^22^	*U*^33^	*U*^12^	*U*^13^	*U*^23^
S1A	0.01653 (10)	0.01155 (8)	0.01764 (9)	−0.00295 (7)	0.00062 (8)	0.00233 (7)
O1A	0.0183 (3)	0.0099 (2)	0.0255 (3)	0.0003 (2)	0.0083 (3)	0.0032 (2)
N1A	0.0178 (4)	0.0107 (3)	0.0176 (3)	−0.0016 (3)	0.0030 (3)	0.0003 (2)
N2A	0.0175 (4)	0.0120 (3)	0.0179 (3)	−0.0015 (3)	0.0046 (3)	−0.0002 (3)
N3A	0.0128 (3)	0.0096 (3)	0.0147 (3)	−0.0002 (2)	0.0010 (3)	0.0000 (2)
N4A	0.0162 (4)	0.0122 (3)	0.0168 (3)	0.0013 (3)	0.0016 (3)	−0.0019 (2)
C1A	0.0129 (4)	0.0119 (3)	0.0141 (3)	−0.0011 (3)	−0.0001 (3)	0.0012 (3)
C2A	0.0146 (4)	0.0098 (3)	0.0156 (3)	−0.0012 (3)	0.0005 (3)	0.0006 (3)
C3A	0.0166 (4)	0.0099 (3)	0.0197 (4)	0.0000 (3)	0.0035 (3)	0.0013 (3)
C4A	0.0146 (4)	0.0114 (3)	0.0157 (3)	−0.0012 (3)	0.0016 (3)	0.0022 (3)
C5A	0.0207 (5)	0.0161 (4)	0.0188 (4)	0.0001 (3)	0.0055 (4)	0.0002 (3)
C6A	0.0237 (5)	0.0241 (4)	0.0176 (4)	−0.0014 (4)	0.0069 (4)	0.0033 (3)
C7A	0.0217 (5)	0.0220 (4)	0.0180 (4)	−0.0039 (4)	0.0027 (4)	0.0068 (3)
C8A	0.0193 (5)	0.0140 (3)	0.0189 (4)	−0.0024 (3)	−0.0007 (3)	0.0053 (3)
C9A	0.0135 (4)	0.0120 (3)	0.0153 (4)	−0.0004 (3)	−0.0009 (3)	0.0018 (3)
C10A	0.0184 (5)	0.0138 (3)	0.0246 (4)	0.0001 (3)	0.0047 (4)	−0.0004 (3)
S1B	0.01796 (11)	0.01668 (9)	0.01883 (10)	−0.00401 (8)	0.00217 (9)	−0.00556 (8)
O1B	0.0195 (3)	0.0124 (2)	0.0176 (3)	−0.0037 (2)	0.0050 (3)	−0.0036 (2)
N1B	0.0191 (4)	0.0138 (3)	0.0186 (3)	0.0014 (3)	−0.0034 (3)	−0.0004 (3)
N2B	0.0212 (4)	0.0110 (3)	0.0202 (3)	0.0016 (3)	−0.0026 (3)	−0.0004 (3)
N3B	0.0142 (4)	0.0101 (3)	0.0141 (3)	0.0004 (2)	−0.0005 (3)	−0.0001 (2)
N4B	0.0175 (4)	0.0150 (3)	0.0206 (3)	0.0016 (3)	−0.0039 (3)	0.0023 (3)
C1B	0.0168 (4)	0.0112 (3)	0.0137 (3)	−0.0004 (3)	0.0020 (3)	−0.0018 (3)
C2B	0.0136 (4)	0.0127 (3)	0.0131 (3)	0.0009 (3)	0.0001 (3)	−0.0012 (3)
C3B	0.0146 (4)	0.0129 (3)	0.0141 (3)	−0.0005 (3)	0.0012 (3)	−0.0009 (3)
C4B	0.0155 (4)	0.0115 (3)	0.0152 (3)	−0.0004 (3)	−0.0013 (3)	−0.0009 (3)
C5B	0.0169 (4)	0.0148 (3)	0.0186 (4)	−0.0018 (3)	0.0020 (3)	−0.0009 (3)
C6B	0.0194 (4)	0.0152 (3)	0.0220 (4)	−0.0033 (3)	−0.0005 (4)	0.0000 (3)
C7B	0.0235 (5)	0.0128 (3)	0.0212 (4)	−0.0021 (3)	−0.0032 (4)	−0.0010 (3)
C8B	0.0212 (5)	0.0142 (3)	0.0163 (4)	0.0013 (3)	−0.0028 (3)	−0.0027 (3)
C9B	0.0153 (4)	0.0143 (3)	0.0132 (3)	0.0009 (3)	−0.0007 (3)	−0.0011 (3)
C10B	0.0222 (5)	0.0212 (4)	0.0178 (4)	−0.0025 (4)	0.0049 (4)	−0.0027 (3)

### Geometric parameters (Å, °)

**Table:**

S1A—C1A	1.6796 (9)	S1B—C1B	1.6771 (10)
O1A—C4A	1.3751 (11)	O1B—C4B	1.3895 (11)
O1A—C3A	1.4141 (11)	O1B—C3B	1.4334 (12)
N1A—C2A	1.3057 (12)	N1B—C2B	1.3086 (12)
N1A—N2A	1.3821 (11)	N1B—N2B	1.3720 (12)
N2A—C1A	1.3401 (12)	N2B—C1B	1.3412 (14)
N2A—H2N1	0.869 (19)	N2B—H2N2	0.894 (18)
N3A—C2A	1.3687 (11)	N3B—C2B	1.3709 (13)
N3A—C1A	1.3740 (12)	N3B—C1B	1.3754 (11)
N3A—N4A	1.4003 (11)	N3B—N4B	1.4023 (11)
N4A—H1N4	0.879 (18)	N4B—H3N4	0.91 (2)
N4A—H2N4	0.832 (18)	N4B—H4N4	0.95 (2)
C2A—C3A	1.4854 (13)	C2B—C3B	1.4888 (12)
C3A—H3AA	0.97	C3B—H3BA	0.97
C3A—H3AB	0.97	C3B—H3BB	0.97
C4A—C5A	1.3901 (14)	C4B—C5B	1.3935 (14)
C4A—C9A	1.4034 (12)	C4B—C9B	1.4054 (13)
C5A—C6A	1.3992 (14)	C5B—C6B	1.4000 (13)
C5A—H5AA	0.93	C5B—H5BA	0.93
C6A—C7A	1.3914 (16)	C6B—C7B	1.3886 (15)
C6A—H6AA	0.93	C6B—H6BA	0.93
C7A—C8A	1.3908 (16)	C7B—C8B	1.3903 (16)
C7A—H7AA	0.93	C7B—H7BA	0.93
C8A—C9A	1.3905 (13)	C8B—C9B	1.3994 (13)
C8A—H8AA	0.93	C8B—H8BA	0.93
C9A—C10A	1.4986 (14)	C9B—C10B	1.5037 (15)
C10A—H10A	0.96	C10B—H10D	0.96
C10A—H10B	0.96	C10B—H10E	0.96
C10A—H10C	0.96	C10B—H10F	0.96
			
C4A—O1A—C3A	116.66 (8)	C4B—O1B—C3B	116.13 (7)
C2A—N1A—N2A	103.87 (7)	C2B—N1B—N2B	104.16 (8)
C1A—N2A—N1A	113.47 (8)	C1B—N2B—N1B	113.71 (8)
C1A—N2A—H2N1	128.2 (12)	C1B—N2B—H2N2	124.2 (12)
N1A—N2A—H2N1	117.4 (12)	N1B—N2B—H2N2	121.1 (12)
C2A—N3A—C1A	108.73 (7)	C2B—N3B—C1B	108.71 (8)
C2A—N3A—N4A	124.09 (8)	C2B—N3B—N4B	124.29 (8)
C1A—N3A—N4A	127.12 (7)	C1B—N3B—N4B	127.00 (8)
N3A—N4A—H1N4	110.4 (12)	N3B—N4B—H3N4	104.0 (11)
N3A—N4A—H2N4	104.0 (12)	N3B—N4B—H4N4	104.5 (12)
H1N4—N4A—H2N4	113.4 (15)	H3N4—N4B—H4N4	110.0 (17)
N2A—C1A—N3A	103.07 (7)	N2B—C1B—N3B	102.92 (8)
N2A—C1A—S1A	129.61 (7)	N2B—C1B—S1B	129.71 (7)
N3A—C1A—S1A	127.31 (7)	N3B—C1B—S1B	127.36 (8)
N1A—C2A—N3A	110.86 (8)	N1B—C2B—N3B	110.50 (8)
N1A—C2A—C3A	127.30 (8)	N1B—C2B—C3B	124.61 (9)
N3A—C2A—C3A	121.84 (8)	N3B—C2B—C3B	124.88 (8)
O1A—C3A—C2A	106.32 (8)	O1B—C3B—C2B	107.54 (8)
O1A—C3A—H3AA	110.5	O1B—C3B—H3BA	110.2
C2A—C3A—H3AA	110.5	C2B—C3B—H3BA	110.2
O1A—C3A—H3AB	110.5	O1B—C3B—H3BB	110.2
C2A—C3A—H3AB	110.5	C2B—C3B—H3BB	110.2
H3AA—C3A—H3AB	108.7	H3BA—C3B—H3BB	108.5
O1A—C4A—C5A	124.21 (8)	O1B—C4B—C5B	123.10 (8)
O1A—C4A—C9A	114.25 (8)	O1B—C4B—C9B	115.43 (8)
C5A—C4A—C9A	121.54 (8)	C5B—C4B—C9B	121.46 (8)
C4A—C5A—C6A	119.23 (9)	C4B—C5B—C6B	119.51 (9)
C4A—C5A—H5AA	120.4	C4B—C5B—H5BA	120.2
C6A—C5A—H5AA	120.4	C6B—C5B—H5BA	120.2
C7A—C6A—C5A	120.08 (10)	C7B—C6B—C5B	119.98 (10)
C7A—C6A—H6AA	120.0	C7B—C6B—H6BA	120.0
C5A—C6A—H6AA	120.0	C5B—C6B—H6BA	120.0
C8A—C7A—C6A	119.67 (9)	C6B—C7B—C8B	119.82 (9)
C8A—C7A—H7AA	120.2	C6B—C7B—H7BA	120.1
C6A—C7A—H7AA	120.2	C8B—C7B—H7BA	120.1
C9A—C8A—C7A	121.58 (9)	C7B—C8B—C9B	121.72 (9)
C9A—C8A—H8AA	119.2	C7B—C8B—H8BA	119.1
C7A—C8A—H8AA	119.2	C9B—C8B—H8BA	119.1
C8A—C9A—C4A	117.87 (9)	C8B—C9B—C4B	117.51 (9)
C8A—C9A—C10A	121.82 (8)	C8B—C9B—C10B	120.86 (9)
C4A—C9A—C10A	120.31 (8)	C4B—C9B—C10B	121.64 (8)
C9A—C10A—H10A	109.5	C9B—C10B—H10D	109.5
C9A—C10A—H10B	109.5	C9B—C10B—H10E	109.5
H10A—C10A—H10B	109.5	H10D—C10B—H10E	109.5
C9A—C10A—H10C	109.5	C9B—C10B—H10F	109.5
H10A—C10A—H10C	109.5	H10D—C10B—H10F	109.5
H10B—C10A—H10C	109.5	H10E—C10B—H10F	109.5
			
C2A—N1A—N2A—C1A	0.60 (11)	C2B—N1B—N2B—C1B	0.20 (12)
N1A—N2A—C1A—N3A	−0.59 (11)	N1B—N2B—C1B—N3B	0.26 (11)
N1A—N2A—C1A—S1A	−179.77 (8)	N1B—N2B—C1B—S1B	−178.52 (8)
C2A—N3A—C1A—N2A	0.35 (10)	C2B—N3B—C1B—N2B	−0.61 (10)
N4A—N3A—C1A—N2A	177.58 (9)	N4B—N3B—C1B—N2B	179.33 (9)
C2A—N3A—C1A—S1A	179.56 (7)	C2B—N3B—C1B—S1B	178.21 (7)
N4A—N3A—C1A—S1A	−3.21 (14)	N4B—N3B—C1B—S1B	−1.85 (14)
N2A—N1A—C2A—N3A	−0.34 (11)	N2B—N1B—C2B—N3B	−0.59 (11)
N2A—N1A—C2A—C3A	179.47 (10)	N2B—N1B—C2B—C3B	−179.27 (9)
C1A—N3A—C2A—N1A	0.00 (11)	C1B—N3B—C2B—N1B	0.79 (11)
N4A—N3A—C2A—N1A	−177.34 (9)	N4B—N3B—C2B—N1B	−179.15 (9)
C1A—N3A—C2A—C3A	−179.83 (9)	C1B—N3B—C2B—C3B	179.47 (9)
N4A—N3A—C2A—C3A	2.84 (14)	N4B—N3B—C2B—C3B	−0.47 (14)
C4A—O1A—C3A—C2A	179.99 (8)	C4B—O1B—C3B—C2B	175.45 (8)
N1A—C2A—C3A—O1A	−16.61 (14)	N1B—C2B—C3B—O1B	90.26 (11)
N3A—C2A—C3A—O1A	163.18 (9)	N3B—C2B—C3B—O1B	−88.24 (11)
C3A—O1A—C4A—C5A	10.15 (15)	C3B—O1B—C4B—C5B	13.09 (13)
C3A—O1A—C4A—C9A	−170.52 (9)	C3B—O1B—C4B—C9B	−167.61 (8)
O1A—C4A—C5A—C6A	−179.92 (10)	O1B—C4B—C5B—C6B	179.26 (9)
C9A—C4A—C5A—C6A	0.80 (16)	C9B—C4B—C5B—C6B	0.00 (15)
C4A—C5A—C6A—C7A	0.55 (17)	C4B—C5B—C6B—C7B	1.03 (16)
C5A—C6A—C7A—C8A	−1.12 (17)	C5B—C6B—C7B—C8B	−1.06 (16)
C6A—C7A—C8A—C9A	0.37 (17)	C6B—C7B—C8B—C9B	0.06 (16)
C7A—C8A—C9A—C4A	0.93 (15)	C7B—C8B—C9B—C4B	0.93 (14)
C7A—C8A—C9A—C10A	−178.93 (10)	C7B—C8B—C9B—C10B	−179.14 (10)
O1A—C4A—C9A—C8A	179.13 (9)	O1B—C4B—C9B—C8B	179.73 (9)
C5A—C4A—C9A—C8A	−1.52 (15)	C5B—C4B—C9B—C8B	−0.96 (14)
O1A—C4A—C9A—C10A	−1.01 (13)	O1B—C4B—C9B—C10B	−0.20 (13)
C5A—C4A—C9A—C10A	178.34 (10)	C5B—C4B—C9B—C10B	179.11 (10)

### Hydrogen-bond geometry (Å, °)

**Table:**

*D*—H···*A*	*D*—H	H···*A*	*D*···*A*	*D*—H···*A*
N4A—H1N4···N4B^i^	0.88 (2)	2.46 (2)	3.2651 (12)	152 (2)
N4A—H2N4···O1B	0.83 (2)	2.53 (2)	3.3560 (11)	171 (2)
N4B—H4N4···S1A^ii^	0.95 (2)	2.72 (2)	3.6167 (10)	157 (2)
N2A—H2N1···S1B^iii^	0.87 (2)	2.30 (2)	3.1665 (9)	174 (2)
N2B—H2N2···N1A^iv^	0.89 (2)	2.18 (2)	3.0589 (11)	166 (2)
C8A—H8AA···S1A^v^	0.93	2.86	3.4537 (10)	123
C3B—H3BB···S1A	0.97	2.86	3.8203 (10)	170

Symmetry codes: (i) −*x*+1, −*y*+1, *z*−1/2; (ii) *x*+1, *y*, *z*; (iii) *x*−1, *y*, *z*; (iv) *x*+1/2, −*y*+3/2, *z*; (v) −*x*+1/2, *y*+1/2, *z*−1/2.
